# Enhancement of Palmarumycin C_12_ and C_13_ Production by the Endophytic Fungus *Berkleasmium* sp. Dzf12 in an Aqueous-Organic Solvent System

**DOI:** 10.3390/molecules201119700

**Published:** 2015-11-12

**Authors:** Yan Mou, Dan Xu, Ziling Mao, Xuejiao Dong, Fengke Lin, Ali Wang, Daowan Lai, Ligang Zhou, Bingyan Xie

**Affiliations:** 1 Department of Plant Pathology, College of Agronomy and Biotechnology, China Agronomy and Biotechnology, Beijing 100193, China; muyan01987@163.com (Y.M.); cauxudan@163.com (D.X.); maoziling2011@163.com (Z.M.); 18874783364@163.com (X.D.); fengkelin.123321aa@foxmail.com (F.L.); wangali526@163.com (A.W.); dwlai@cau.edu.cn (D.L.); 2 Institute of Vegetables and Flowers, Chinese Academy of Agricultural Sciences, Beijing 100081, China

**Keywords:** endophytic fungus, *Berkleasmium* sp. Dzf12, *Dioscorea zingiberensis*, palmarumycin C_12_, palmarumycin C_13_, production, aqueous-organic solvent system, butyl oleate, liquid paraffin

## Abstract

The endophytic fungus *Berkleasmium* sp. Dzf12, isolated from *Dioscorea zingiberensis*, was found to produce palmarumycins C_12_ and C_13_ which possess a great variety of biological activities. Seven biocompatible water-immiscible organic solvents including *n*-dodecane, *n*-hexadecane, 1-hexadecene, liquid paraffin, dibutyl phthalate, butyl oleate and oleic acid were evaluated to improve palmarumycins C_12_ and C_13_ production in suspension culture of *Berkleasmium* sp. Dzf12. Among the chosen solvents both butyl oleate and liquid paraffin were the most effective to improve palmarumycins C_12_ and C_13_ production. The addition of dibutyl phthalate, butyl oleate and oleic acid to the cultures of *Berkleasmium* sp. Dzf12 significantly enhanced palmarumycin C_12_ production by adsorbing palmarumycin C_12_ into the organic phase. When butyl oleate was fed at 5% (*v*/*v*) in medium at the beginning of fermentation (day 0), the highest palmarumycin C_12_ yield (191.6 mg/L) was achieved, about a 34.87-fold increase in comparison with the control (5.3 mg/L). *n*-Dodecane, 1-hexadecene and liquid paraffin had a great influence on the production of palmarumycin C_13_. When liquid paraffin was added at 10% (*v*/*v*) in medium on day 3 of fermentation, the palmarumycin C_13_ yield reached a maximum value (134.1 mg/L), which was 4.35-fold that of the control (30.8 mg/L). Application of the aqueous-organic solvent system should be a simple and efficient process strategy for enhancing palmarumycin C_12_ and C_13_ production in liquid cultures of the endophytic fungus *Berkleasmium* sp. Dzf12.

## 1. Introduction

Plant endophytic fungi are micro-organisms which colonize the interior parts of host plants, typically causing no apparent symptoms of pathogenesis and being well recognized as a promising source of bioactive compounds [1,2,3]. Up to now, a large number of endophytic fungi have been isolated and identified from plants, such as *Fusarium oxysporum*, *Aspergillus niger* and *Alternaria tenuissima* for the production of podophyllotoxin, naphtho-*γ*-pyrones and tricycloalternarene derivatives, respectively [4,5,6].

**Figure 1:**
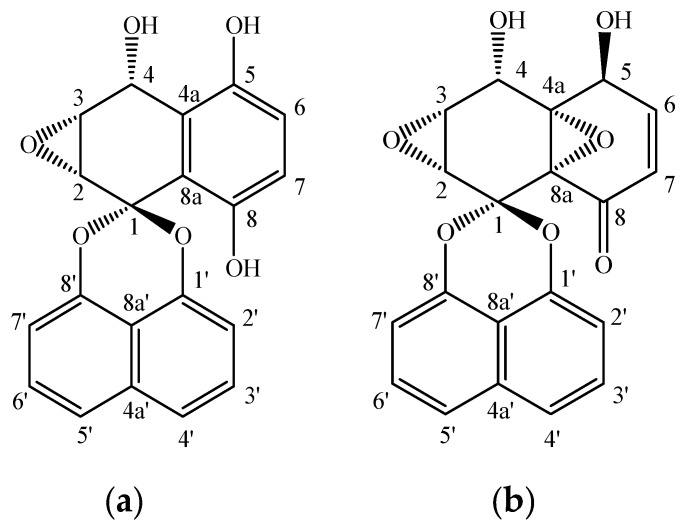
Chemical structures of palmarumycins C_12_ (**a**) and C_13_ (**b**).

Spirobisnaphthalenes are a large class of secondary metabolites found in various fungal species which possess a wide range of biological activities such as antibacterial, antifungal, and anti-tumor activities and enzyme-inhibitory properties with applications in agriculture, medicine and the food industry [7,8]. Palmarumycins C_12_ and C_13_ (Figure 1) belong to the deoxypreussomerin type of spirobisnaphthalenes, consisting of a 1,8-dihydroxynaphthalene-derived spiroketal unit linked to a second oxidized naphthalene moiety, show a great variety of biological activities. Palmarumycin C_12_ showed antifungal activity on *Ustilago violacea* and *Eurotium repens* [9]. Palmarumycin C_13_ (also variously named Sch 53514, diepoxin ζ and cladospirone bisepoxide) exhibited obvious antibacterial activity on *Bacillus megaterium*, *Xanthomonas vesicatoria* and *Escherichia coli* [10,11], antifungal activity on *Magnaporthe oryzae* [12], anti-algae activity on *Chlorella fusca* [9,13], antitumor activity on human fibrosarcoma cells [14,15], and inhibitory activity on phospholipase D (PLD) [13]. In comparison, palmarumycin C_13_ with more and more varied biological activities is considered more valuable than palmarumycin C_12_. Moreover, it is generally accepted that palmarumycin C_12_ is the precursor of palmarumycin C_13_ [16]. Due to their potential applications, commercial demand for these compounds could be expected to increase in the future, so it is of great importance to develop an efficient way to produce palmarumycin C_12_ and C_13_ economically.

Various biological, chemical or physical strategies such as medium optimization [17], metal ions addition [18], ultraviolet irradiation [19], ultrasonic treatment [20], immobilization [21], two-phase culture [22], and co-culture [23,24] have been employed to improve the productivity of plant and microbial metabolites for their industrial exploitation [25]. Among these attempts, the aqueous-organic two-phase culture system, which employs a partitioning system to enhance the release of the metabolites from the cells, has been considered as a practical strategy [26]. The organic phases can fall into two categories depending on their functions: one class like the glycerol derivative Miglyol, silicone fluid [27,28], tricaprylin [29], dioctyl phthalate [30] and isobutanol [31] redirect extracellular products into a second phase to ease the extraction and purification of the secondary metabolites by *in situ* removal, and afford higher yields by removing the metabolites from the aqueous medium to decrease the feedback inhibition on cell metabolism [32]. Another class of organic phases includes the hydrocarbons such as *n*-hexane, *n*-heptane, *n*-dodecane and *n*-hexadecane, which play an important role as oxygen vectors or carbon sources to increase metabolite production [33,34,35]. The addition of these organic solvents may provoke a significant increase in the transfer rate from the gas phase to the microorganisms due to their high oxygen solubility [36]. The oxygen solubility in these compounds is about 15 to 20 times higher than in water, which significantly increases the dissolved oxygen (DO) concentration in the fermentation medium and, as a consequence, enhances cell growth rates and the yields of targeted products [37,38]. The advantages of these biocompatible water-immiscible organic phases are effective physical accumulation, oxygen solubility, autoclavability, lack of toxicity and low cost. Furthermore, the selection of an appropriate second organic phase is an important step for increasing secondary metabolite production. The solvent is chosen according to its chemical properties for facilitating the selective migration of the desired product from the feed phase and for ensuring the rapid disengagement of the two phases after contact [36]. The addition time, concentration and volume fraction of the organic phase also have strong impacts on the production of secondary metabolites in aqueous–organic two-phase culture systems [39,40].

Twenty two spirobisnaphthalenes can be isolated from the endophytic fungus *Berkleasmium* sp. Dzf12 derived from the healthy rhizomes of the medicinal plant *Dioscorea zingiberensis* [9,11]. Among them, the palmarumycins C_12_ and C_13_, the two main spirobisnaphthalenes, were found to be released from the intracellular to the extracellular medium. Some attempts such as medium optimization as well as the application of metal ions, macroporous resins and elicitors have been made to effectively improve palmarumycin C_12_ and C_13_ production [18,41,42,43,44]. So far, there have been no previous reports on the enhancement of palmarumycin C_12_ and C_13_ production by the endophytic fungus *Berkleasmium* sp. Dzf12 in an aqueous-organic two-phase culture system. Theaim of the present work was therefore to explore the effects of seven biocompatible organic solvents, including *n*-dodecane, *n*-hexadecane, 1-hexadecene, liquid paraffin, dibutyl phthalate (DBP), butyl oleate and oleic acid on the production of palmarumycin C_12_ and C_13_ in liquid cultures of the endophytic fungus *Berkleasmium* sp. Dzf12. The most effective solvent with its addition time and concentration could then be selected for application in the aqueous-organic two-phase culture process for maximizing palmarumycin C_12_ and C_13_ production.

## 2. Results and Discussion

### 2.1. Effects of the Organic Solvents on Mycelial Growth of Berkleasmium sp. Dzf12 

**Figure 2:**
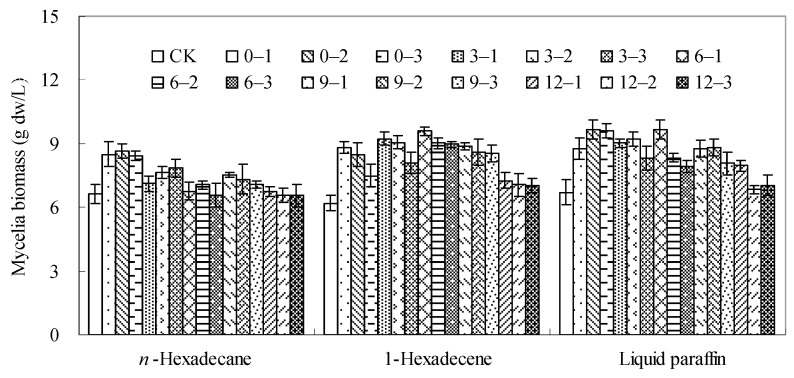
Effects of *n*-hexadecane, 1-hexadecene and liquid paraffin on mycelia growth in liquid culture of *Berkleasmium* sp. Dzf12. The organic solvents were applied at 5%, 10% and 15% on days 0, 3, 6, 9 and 12 of culture, respectively. The period of culture lasted for 15 days. “CK” means the control without any organic solvents. 0-1, 0-2 and 0-3 mean that the organic solvent was applied on day 0 at 5%, 10% and 15%, respectively. 3-1, 3-2 and 3-3 mean that the organic solvent was applied on day 3 at 5%, 10% and 15%, respectively. 6-1, 6-2 and 6-3 mean that the organic solvent was applied on day 6 at 5%, 10% and 15%, respectively. 9-1, 9-2 and 9-3 mean that the organic solvent was applied on day 9 at 5%, 10% and 15%, respectively. 12-1, 12-2 and 12-3 mean that the organic solvent was applied on day 12 at 5%, 10% and 15%, respectively. The error bars represent standard deviations from three independent samples.

Seven organic solvents including *n*-dodecane, *n*-hexadecane, 1-hexadecene, liquid paraffin, dibutyl phthalate (DBP), butyl oleate and oleic acid were respectively added to the culture broth of *Berkleasmium* sp. Dzf12 at final concentrations of 5%, 10% and 15% on days 0, 3, 6, 9 and 12 of culture. The effects of these organic solvents on mycelial growth were determined after the mycelia were cultured for 15 days. As shown in Figure 2 and Table S1, all the organic solvents used were nontoxic to the mycelia. This trend was consistent with the general rule that a biocompatible organic solvent should have a value of log *p* > 4 (Table 1) [45]. Among them, *n*-dodecane, DBP, butyl oleate and oleic acid showed no effect on the mycelia growth, and *n*-hexadecane was slightly beneficial, and 1-hexadecene and liquid paraffin were the most beneficial to the mycelia growth. Correspondingly, *n*-hexadecane, 1-hexadecene and liquid paraffin stimulated the mycelia growth compared to the control without solvent addition, and the mycelia biomass increased from 0% to 33%, 13% to 55% and 2% to 44%, respectively. When *n*-hexadecane was fed at 10% in medium at the time of inoculation (day 0), the mycelia biomass was as much as 8.7 g dw/L, about 33% increase in comparison with the control (6.6 g dw/L). When 1-hexadecene was applied to the medium at 5% on day 6 of culture, the mycelia biomass reached 9.6 g dw/L, about a 55% increase in comparison with the control (6.2 g dw/L). With either 10% of liquid paraffin fed on day 3 or 5% of liquid paraffin fed on day 6, the highest mycelia biomass (9.7 or 9.7 g dw/L) was obtained, about 44% increase in comparison with the control (6.7 g dw/L). According to the trend shown in Figure 2, the higher concentration of the organic solvents depressed the mycelia growth rate. The possible reasons may be the interfacial deactivation of the cells, the absorption of hydrophobic nutrients, particularly the organic nutrients, and the interference with nutrient and oxygen transfer to the cells [46].

In our results, although the log *p* values of 1-hexadecene and liquid paraffin were uncertain, they were found to be the most effective organic solvents for *Berkleasmium* sp. Dzf12 mycelia growth. Combined with the chemical properties of the employed organic solvents, it can be deduced that both log *p* values of 1-hexadecene and liquid paraffin might be greater than 4 for their positive effects on mycelia growth of *Berkleasmium* sp. Dzf12.

**Table 1:** Physicochemical properties of the employed organic solvents.

Organic Solvent	Molecular Weight (Da)	Density (g/mL)	Boiling Point (°C)	Log *p*
*n*-Dodecane	170	0.75	216	6.6
*n*-Hexadecane	226	0.89	287	8.8
1-Hexadecene	224	0.78	285	none
Liquid paraffin	150–250	0.83–0.86	185–250	none
Dibutyl phthalate	278	1.05	340	5.4
Butyl oleate	339	0.88	227–228	9.8
Oleic acid	282	0.89	360	7.7

Note: log *p* was the logarithm of partition coefficient of the organic solvent in octanol/water. Liquid paraffin is a mixture of C_16_–C_20_ alkanes.

### 2.2. Effects of the Organic Solvents on Palmarumycin C_12_ Production on Berkleasmium sp. Dzf12

**Figure 3:**
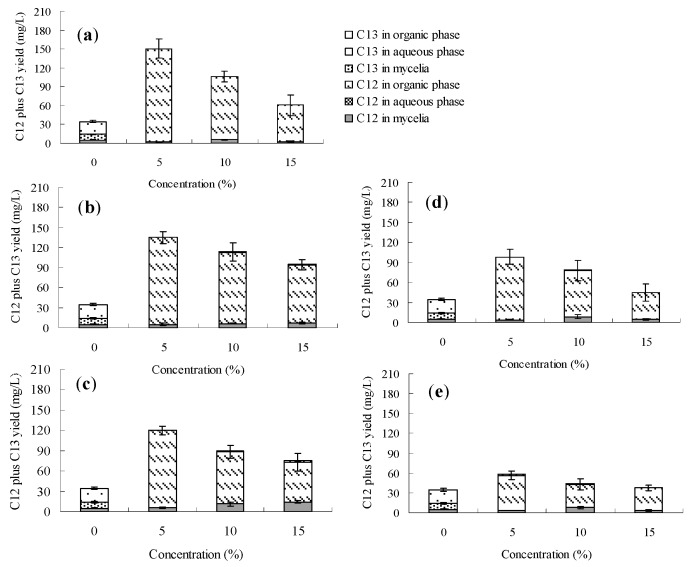
Effects of dibutyl phthalate on palmarumycin production in liquid culture of *Berkleasmium* sp. Dzf12. Dibutyl phthalate was applied at 0%, 5%, 10% and 15% on days 0 (**a**); 3 (**b**); 6 (**c**); 9 (**d**) and 12 (**e**) of culture, respectively. The period of culture lasted for 15 days. “C12” means palmarumycin C_12_, “C13” means palmarumycin C_13_. The error bars represent standard deviations from three independent samples.

**Figure 4:**
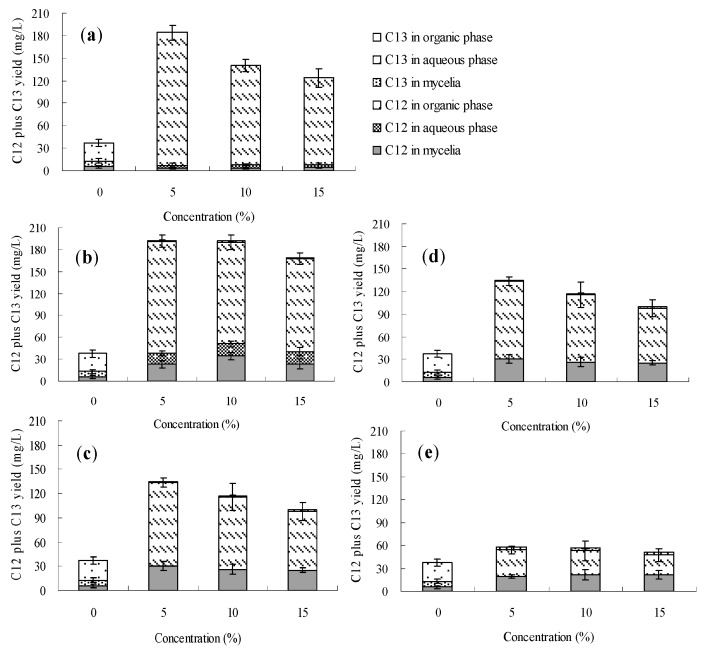
Effects of butyl oleate on palmarumycin production in liquid culture of *Berkleasmium* sp. Dzf12. Butyl oleate was applied at 0%, 5%, 10% and 15% on days 0 (**a**); 3 (**b**); 6 (**c**); 9 (**d**) and 12 (**e**) of culture, respectively. The period of culture lasted for 15 days. “C12” means palmarumycin C_12_, “C13” means palmarumycin C_13_. The error bars represent standard deviations from three independent samples.

**Figure 5:**
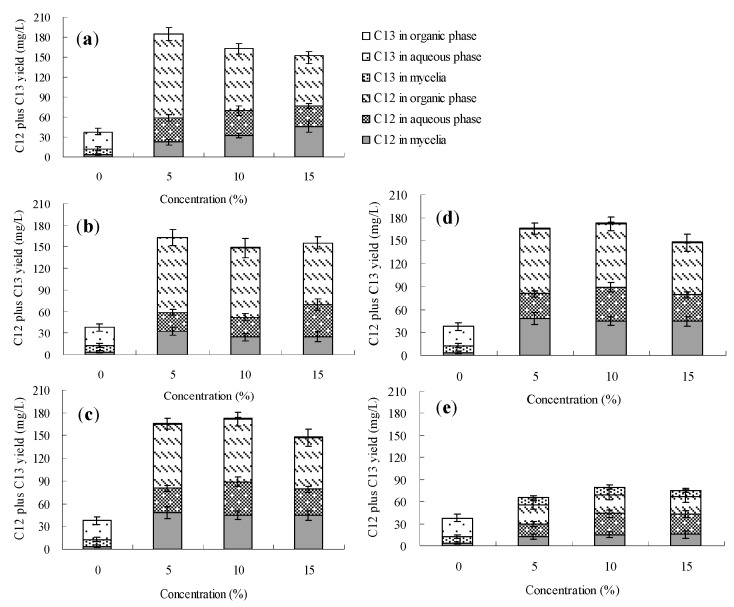
Effects of oleic acid on palmarumycin production in liquid culture of *Berkleasmium* sp. Dzf12. Oleic acid was applied at 0%, 5%, 10% and 15% on days 0 (**a**); 3 (**b**); 6 (**c**); 9 (**d**) and 12 (**e**) of culture, respectively. The period of culture lasted for 15 days. “C12” means palmarumycin C_12_, “C13” means palmarumycin C_13_. The error bars represent standard deviations from three independent samples.

The palmarumycin C_12_ biosynthesis of *Berkleasmium* sp. Dzf12 in normal culture (without addition of any organic solvent) was always found to occur in the mycelia, and the maximum palmarumycin C_12_ yield was about 6.5 mg/L. Among the seven solvents, only dibutyl phthalate (DBP), butyl oleate and oleic acid could transfer extracellular palmarumycins (*i.e*., in this case palmarumycin C_12_) into the second organic phase throughout the course of the fermentation. The effects of DBP, butyl oleate and oleic acid on palmarumycin production were examined at different concentrations (5%, 10% and 15%, *v*/*v*) and different addition time (days 0, 3, 6, 9 and 12). Figure 3, Figure 4 and Figure 5 show respectively that palmarumycin C_12_ yield in the two-phase culture with any of the three organic solvents was always higher than in the single-phase control culture, and palmarumycin C_13_ was hardly detected in the mycelia, liquid medium or any organic solvent. The results are consistent with the deduction that palmarumycin C_12_ is the precursor of palmarumycin C_13_ [8,16]. Palmarumycin C_12_ was absorbed from the mycelia into the organic solvents, which could prevent its transformation into palmarumycin C_13_. Absorption of palmarumycin C_12_ from the heterogeneous system into the organic solvents also reduced product feedback inhibition. Whether the low production of palmarumycin C_13_ is due to either palmarumycin C_12_ absorption by the organic solvents or enzyme inhibition by the solvents needs further study. HPLC analysis of palmarumycin production in liquid culture of *Berkleasmium* sp. Dzf12 with oleic acid as the water-immiscible organic solvent is shown in Figure S1.

Palmarumycin C_12_ yield was increased significantly when the concentration of DBP, butyl oleate and oleic acid was set at 5% (*v*/*v*). However, if the concentration of DBP, butyl oleate or oleic acid was too high, palmarumycin C_12_ production was negatively affected. Meanwhile, the solvent addition time was also an important factor affecting palmarumycin production in the two-phase culture system. Our results suggested that the optimal addition time was during the early stages of the fermentation process. The palmarumycin C_12_ yield with the late (day 12) solvent addition was lower than that obtained with early (days 0–6) addition but still much higher than the control. When the organic solvent was added during the late fermentation phase (day 12), it only had a short period of time to affect the palmarumycin C_12_ production. The short-term effect suggested that the solvent had a fast-acting stimulatory effect on biosynthesis of secondary metabolites [46].

With different addition time and concentrations, 80%–99%, 54%–96% and 36%–68% of palmarumycin C_12_ were detected in the organic solvents DBP, butyl oleate and oleic acid, respectively (Figure 3, Figure 4 and Figure 5 and Tables S2–S4). The absorption ability of DBP was found to be optimum in that palmarumycin C_12_ was rarely detected in mycelia or aqueous liquid medium. When DBP, butyl oleate and oleic acid were applied to the medium at 5% (*v*/*v*) on day 0, the maximal yields of palmarumycin C_12_ were reached 150.6, 192.5 and 184.7 mg/L, which were 30.31-, 34.87- and 46.98-fold higher in comparison with that of control, respectively. The reason for this is possibly that palmarumycin yields in normal culture often show large deviations (up to 25% of the mean) between the repeat runs, but the overall trends are clearly reproducible. These results suggested that butyl oleate was the most desirable solvent for palmarumycin C_12_ production in the aqueous-organic culture system of *Berkleasmium* sp. Dzf12. The satisfactory biocompatibility with the mycelia (log *p* = 9.8) and large partition coefficient for palmarumycin C_12_ might contribute to palmarumycin C_12_ production by addition of butyl oleate [45].

### 2.3. Effects of the Organic Solvents on Palmarumycin C_13_ Production on Berkleasmium sp. Dzf12

**Figure 6:**
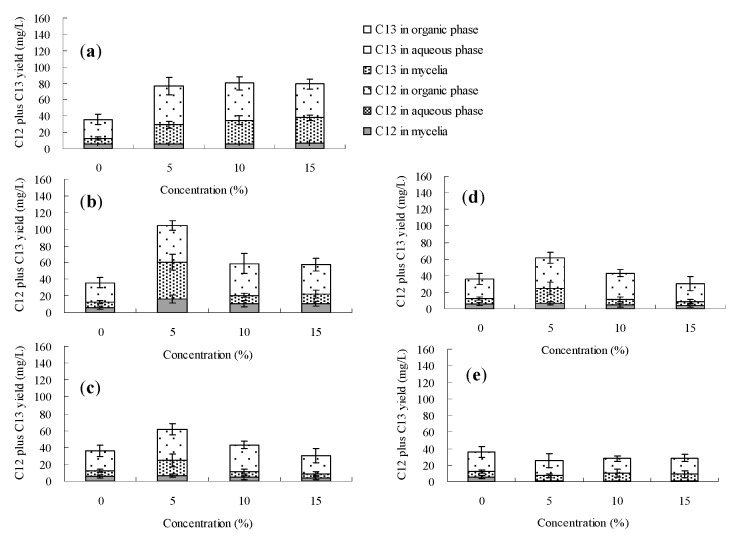
Effects of *n*-dodecane on palmarumycin production in liquid culture of *Berkleasmium* sp. Dzf12. *n*-Dodecane was applied at 0%, 5%, 10% and 15% on days 0 (**a**); 3 (**b**); 6 (**c**); 9 (**d**) and 12 (**e**) of culture, respectively. The period of culture lasted for 15 days. “C12” means palmarumycin C_12_, “C13” means palmarumycin C_13_. The error bars represent standard deviations from three independent samples.

**Figure 7:**
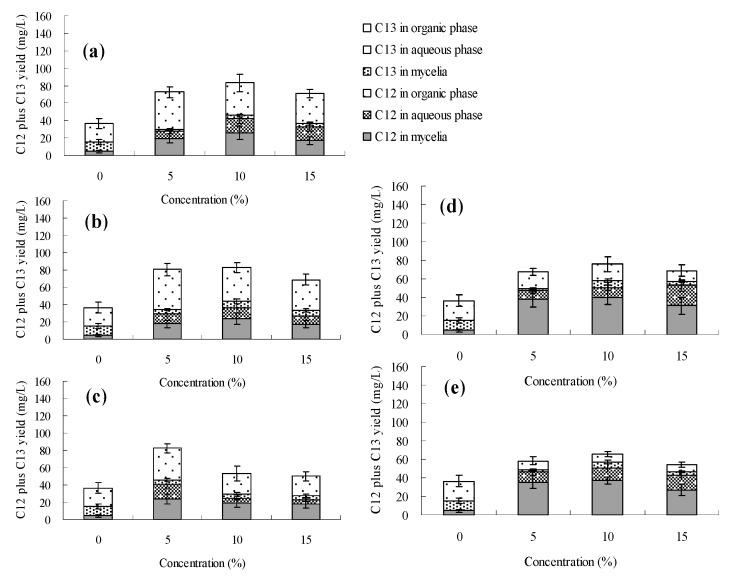
Effects of *n*-hexadecane on palmarumycin production in liquid culture of *Berkleasmium* sp. Dzf12. *n*-Hexadecane was applied at 0%, 5%, 10% and 15% on days 0 (**a**); 3 (**b**); 6 (**c**); 9 (**d**) and 12 (**e**) of culture, respectively. The period of culture lasted for 15 days. “C12” means palmarumycin C_12_, “C13” means palmarumycin C_13_. The error bars represent standard deviations from three independent samples.

**Figure 8:**
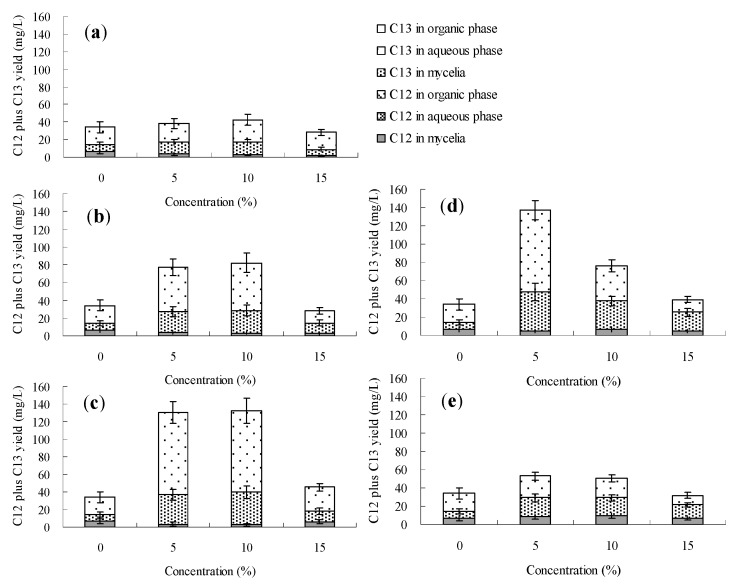
Effects of 1-hexadecene on palmarumycin production in liquid culture of *Berkleasmium* sp. Dzf12. 1-Hexadecene was applied at 0%, 5%, 10% and 15% on days 0 (**a**); 3 (**b**); 6 (**c**); 9 (**d**) and 12 (**e**) of culture, respectively. The period of culture lasted for 15 days. “C12” means palmarumycin C_12_, “C13” means palmarumycin C_13_. The error bars represent standard deviations from three independent samples.

**Figure 9:**
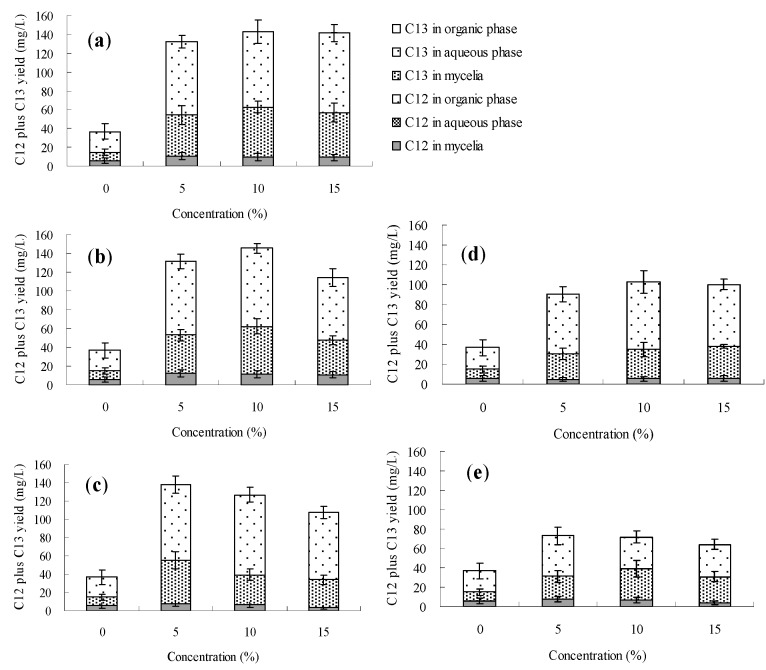
Effects of liquid paraffin on palmarumycin production in liquid culture of *Berkleasmium* sp. Dzf12. Liquid paraffin was applied at 0%, 5%, 10% and 15% on days 0 (**a**); 3 (**b**); 6 (**c**); 9 (**d**) and 12 (**e**) of culture, respectively. The period of culture lasted for 15 days. “C12” means palmarumycin C_12_, “C13” means palmarumycin C_13_. The error bars represent standard deviations from three independent samples.

The effects of *n*-dodecane, *n*-hexadecane, 1-hexadecene and liquid paraffin on palmarumycin production in *Berkleasmium* sp. Dzf12 liquid culture are presented in Figure 6, Figure 7, Figure 8 and Figure 9 and Tables S5–S8. It was observed that addition of the four organic solvents at different addition times and various concentrations could improve palmarumycin production to a certain extent. Meanwhile the various short-chain hydrocarbons exhibited different effects on palmarumycin production. Although palmarumycin C_12_ or C_13_ could not be absorbed into the added organic solvents (*n*-dodecane, *n*-hexadecane, 1-hexadecene and liquid paraffin), palmarumycin C_12_ or C_13_ production was significantly increased under some conditions. One reason may be that these liquid hydrocarbons could increase the oxygen transfer rate from the gas phase to the microorganisms without the need for extra energy supply [47]. Another reason may be that they could act as an indirect inducer through the absorption of the produced pigments, diminishing the negative feedback of accumulation by acting as a sink for these products [26].

Based on the results shown in Figure 6 and Table S5, the addition time of *n*-dodecane affected the palmarumycin C_13_ production substantially. At the beginning of the fermentation (day 0–6), palmarumycin C_13_ yield was improved obviously by the addition of *n*-hexadecane (especially at 5%, *v*/*v*). With 5%~15% (*v*/*v*) of *n*-dodecane fed on day 9 or 12, it was found that palmarumycin C_13_ yield was almost unchanged during the course of fermentation. Meanwhile, as the three-day-old cultures treated with 5% (*v*/*v*) of *n*-dodecane, palmarumycin C_13_ reached an optimal production (87.8 mg/L). Figure 7 and Table S6 indicated that the addition of *n*-hexadecane slightly improved palmarumycins C_12_ and C_13_ production at the same time. Production of palmarumycin C_13_ was obviously improved on either day 0 or day 3 of *n*-hexadecane addition, while improvement of palmarumycin C_12_ production mainly focused on either day 9 or day 12 of *n*-hexadecane addition. As shown in Figure 8 and Table S7, the concentrations and addition time of 1-hexadecene had prominently influenced on palmarumycin C_13_ production. When the cultures were fed with 5%–10% of 1-hexadecene on day 6 or 5% of 1-hexadecene on day 9, the highest yield of palmarumycin C_13_ production (about 130 mg/L) was achieved, which was about 3.8-fold higher than the control (27.6 mg/L). Liquid paraffin, a mixture of heavier alkanes containing 16 to 20 carbons, was found to be the most effective organic solvent for palmarumycin C_13_ production (Figure 9 and Table S8). With 10% of liquid paraffin added on day 3, the total palmarumycins production (palmarumycins C_12_ yield plus palmarumycin C_13_ yield) by *Berkleasmium* sp. Dzf12 was improved to reach 145.3 mg/L, which was about 3.94-fold of control yield (36.9 mg/L). The improvement of palmarumycin production may be related to the fact that liquid paraffin could act as an active intermediate in the oxygen transport from gas phase to the aqueous phase throughout the course of the fermentation.

Among the seven organic solvents used in this study, dibutyl phthalate (DBP), butyl oleate and oleic acid, belonging to the first category of the organic solvents, were effective for the production of palmarumycin C_12_. *n*-Dodecane, *n*-hexadecane, 1-hexadecene, and liquid paraffin, belonging to the second category, were effective for the production of palmarumycin C_13_. It is possible that these hydrocarbons (*n*-dodecane, *n*-hexadecane, 1-hexadecene and liquid paraffin) may play a role as oxygen vectors to stimulate transformation of palmarumycin C_12_ to palmarymycin C_13_ [35,48,49]. Dibutyl phthalate (DBP), butyl oleate and oleic acid have no function as oxygen vectors, and just absorbed the palmarumycin C_12_ released from the intracellular to the extracellular medium. As palmarumycin C_13_ is more valuable than palmarumycin C_12_, addition of the organic solvents belonging to the second category (*i.e*., liquid paraffin) could be used for the production of palmarumycin C_13_.

Wu *et al.* [50] reported that the taxol (paclitaxel) yield was improved 7- to 9-fold in *Taxus chinensis* cell cultures with dibutyl phthalate (DBP) (11%, *v*/*v*) used as a second phase, and both the extracellular taxol ratio and taxol release were significantly increased. According to Wei *et al.* [39], oleic acid and DBP were proved to be suitable organic solvents for the accumulation of taxol in *T. chinensis* cell cultures. The optimal volumetric percentage of organic solvents was found to be around 8% (*v*/*v*), and the favorable time was at the exponential phase of cell growth. Li *et al.* tested several kinds of organic solvents included butyl oleate, *n*-hexadecane and liquid paraffin with cell suspension cultures of *Paecilomyces verticillatus* GZ, 8% (*v*/*v*) of *n*-hexadecane was introduced into shake flask culture at the beginning of fermentation, the cordycepin production of *P. verticillatus* was 224% more than the control [51]. *n*-Dodecane has been used as oxygen-vector for improvement of citric acid production by *Aspergillus niger*, increasing the citric acid accumulation by a factor of 1.4-fold [36]. In addition, when 10% (*v*/*v*) of 1-hexadecene was added on day 6 of culture, the yields of palmarumycin C_2_ and C_3_ were about 40- and 60-fold higher, respectively, in comparison with those of the control in liquid culture of *Berkleasmium* sp. Dzf12 [52]. In this study, we employed different types of organic solvents as the second phase for the accumulation of palmarumycins C_12_ and C_13_ in liquid culture of *Berkleasmium* sp. Dzf12, and the different action mechanism of these organic solvents might result in the differences in palmarumycin C_12_ or C_13_ production.

## 3. Experimental Section

### 3.1. Endophytic Fungus and Culture Conditions

The endophytic fungus *Berkleasmium* sp. Dzf12 was isolated from healthy rhizomes of medicinal plant *Dioscorea zingiberensis* C.H. Wright (Dioscoreaceae) [9]. It was maintained on potato-dextrose-agar (PDA) slants and stored at 4 °C and subcultured every other month. Details on the culture and inoculation procedures were previously reported [18]. Based on the general production characteristics of secondary metabolites, a two-stage culture process, including seed and production culture, was applied. Four mycelia disks (about 5 mm) were transferred into each 300-mL Erlenmeyer flask containing 100 mL of seed medium (potato dextrose broth). After 4 days of continuous culture, the seed suspension cultures were inoculated at ratio of 3% (*v*/*v*) in 150-mL Erlenmeyer flasks each containing 30 mL of production medium for 15 days. One liter of production medium contained glucose 40 g, peptone 10 g, KH_2_PO_4_ 1 g, MgSO_4_·7H_2_O 0.5 g and FeSO_4_·7H_2_O 0.05 g, pH 6.5. The media were sterilized at 121 °C for 20 min, and the flasks were incubated at 25 °C and 150 rpm on a rotary shaker in darkness.

### 3.2. Organic Solvents and Two-phase Culture

Seven water-immiscible organic solvents including *n*-dodecane (99.0% purity), *n*-hexadecane (99.0% purity), 1-hexadecene (94.0% purity), liquid paraffin (98.0% purity, composed of C_14_–C_16_ *n*-alkanes), dibutyl phthalate (DBP, 99.0% purity), butyl oleate (98.5% purity) and oleic acid (99.0% purity) purchased from Alfa Aesar (a branch of Johnson Matthey Company, London, UK) were tested in the aqueous-organic two-phase culture system. Their physicochemical properties are listed in Table 1. The log *p* value is an index for selecting biocompatible organic solvents, and in general, an organic solvent having a log *p* greater than 4.0 should be nontoxic to the mycelia [45]. *p* is the partition coefficient of a compound over a standard octanol/water two-phase system, and log *p* may be considered as a measure of solvent polarity (or hydrophobicity) [46]. These organic solvents were autoclaved at 121 °C for 20 min and then added to culture flasks at final concentrations of 5%, 10% and 15% (*v*/*v*), respectively. All experiments were carried out with the additives added at the beginning (0 day), 3 days, 6 days, 9 days and 12 days of fermentation culture, respectively. The period of culture lasted for 15 days. Each experiment was carried out at least three times under the identical conditions.

### 3.3. Measurement of Mycelia Biomass and Palmarumycins C_12_ and C_13_ Yield

The mycelia of the endophytic fungus *Berkleasmium* sp. Dzf12 were separated from the fermentation broth by filtering under vacuum through a pre-weighed filter paper. It was then washed three times with distilled water, dried in an oven at 50–55 °C to a constant dry weight, then the cell dry weight (dw) was obtained.

Palmarumycin extraction and quantification in the samples were based on the methods as described previously [18]. The structures of palmarumycins C_12_ and C_13_ are shown in Figure 1. For palmarumycin C_12_ and C_13_ analysis in mycelia, 50.0 mg of dry mycelia powder was deposited into a vial with 5 mL of methanol-chloroform (9:1, *v*/*v*), and then subjected to ultrasonic treatment (three times, 60 min each). After removal of the solid by filtration, the filtrate was evaporated to dryness and re-dissolved in 1 mL of methanol. For palmarumycin C_12_ and C_13_ analysis in broth, 3 mL of the culture broth without mycelia was evaporated to dryness and extracted with 5 mL of methanol-chloroform (9:1, *v*/*v*) in an ultrasonic bath, and the liquid extract was then evaporated to dryness and re-dissolved in 1 mL of methanol. For analysis of palmarumycins C_12_ and C_13_ in the organic phase, the samples were directly dissolved in methanol, chloroform or acetone (*i.e.*, *n*-dodecane, *n*-hexadecane, 1-hexadecene and liquid paraffin were dissolved in chloroform; dibutyl phthalate (DBP) and oleic acid in methanol; and butyl oleate in acetone) and filtered with a 0.22 µm nylon filter. All samples were analyzed using an HPLC instrument consisting of two LC-20AT solvent delivery units, an SIL-20A autosampler, an SPD-M20A photodiode array detector, and CBM-20Alite system controller (Shimadzu, Kyoto, Japan), and a reversed-phase Ultimate TM XB C_18_ column (4.6 × 250 mm, 5 μm, Welch Materials, Inc., Ellicott, MD, USA). The HPLC column was used with an isocratic mobile phase of MeOH–H_2_O (50:50, *v*/*v*) in 20 min at a flow rate of 1.0 mL/min. The temperature was maintained at 40 °C, and UV detection at 226 nm. The sample injection volume was 10 μL. The LC-solution multi-PDA workstation was employed to acquire and process chromatographic data. Palmarumycins C_12_ and C_13_ were detected and quantified with the standards prepared according to the previous method [9,43]. The regression equation of palmarumycin C_12_ was *Y* = 121295.5*X* + 175236.8 (*R* = 0.9997), and that of palmarumycin C_13_ was *Y* = 121362.5*X* + 256167.5 (*R* = 0.9997), where *Y* is the peak area, *X* is quality (μg) of the sample injected for each time, and *R* is the correlation coefficient.

### 3.4. Statistical Analysis

All the tests were carried out in triplicate, and the results were represented by their mean values and standard deviations (SD). The data were submitted to analysis of variance (one-way ANOVA) to detect significant differences by PROC ANOVA of SAS version 8.2. The term significant has been used to denote the differences for which *p* ≤ 0.05.

## 4. Conclusions

In this study, we report for the first time the enhancement of palmarumycin C_12_ and C_13_ production by the endophytic fungus *Berkleasmium* sp. Dzf12 in an aqueous-organic solvent system. The effects of seven water-immiscible organic solvents including *n*-dodecane, *n*-hexadecane, 1-hexadecene, liquid paraffin, dibutyl phthalate (DBP), butyl oleate and oleic acid on palmarumycin C_12_ and C_13_ production were evaluated by adding them to liquid cultures of the endophytic fungus *Berkleasmium* sp. Dzf12. All the organic solvents had positive effects on the enhancement of palmarumycin C_12_ or C_13_ production during the fermentation process though their action modes might be different. The biocompatibility (log *p* value) of each organic solvent along with its concentration in the culture medium, and addition time to the culture were important factors affecting palmarumycin C_12_ or C_13_ yield in the aqueous-organic two-phase culture process [46]. In the presence of dibutyl phthalate (DBP), butyl oleate and oleic acid additives, palmarumycin C_12_ production was increased dramatically, and butyl oleate had been proved to be the most efficient organic solvent. With a final concentration of 5% (*v*/*v*) butyl oleate fed at the beginning of fermentation (day 0), the highest palmarumycin C_12_ yield (191.6 mg/L) was achieved, about 34.87-fold increase in comparison with the control of 5.3 mg/L. In addition, *n*-dodecane, *n*-hexadecane, 1-hexadecene and liquid paraffin obviously enhanced palmarumycin C_13_ production. Among them, liquid paraffin was the most effective. When liquid paraffin was added at 10% on day 3, palmarumycin C_13_ yield was improved to reach 134.1 mg/L, which was about 4.39-fold of control (30.8 mg/L). As palmarumycin C_13_ was more valuable than palmarumycin C_12_, addition of the organic solvents belonging to the second category could be beneficial to the production of palmarumycin C_13_.

The organic solvents used in this study were non-toxic, biologically inert, readily available commercially, inexpensive, easily separable from the medium, and reusable [53]. They have great potential for the efficient production of palmarumycin C_12_ or C_13_ in aqueous-organic solvent *Berkleasmium* sp. Dzf12 culture systems. However, the effects of organic solvents on mycelia growth and palmarumycin biosynthesis are still not well understood, and the more investigations are required to study in detail the solvent-mycelia and solvent-medium interactions.

## Supplementary Materials

Supplementary materials can be accessed at: http://www.mdpi.com/1420-3049/20/11/19700/s1.
