# Supplementary material for: Enhancement of Palmarumycin C12 and C13 Production by the Endophytic Fungus Berkleasmium sp. Dzf12 in an Aqueous-Organic Solvent System
Source: Molecules. 2015 Nov 12;20(11):20320–33. doi: 10.3390/molecules201119700 (PMC6331930; doi:10.3390/molecules201119700)
Supplement: Supplementary file 1 [file molecules-20-19700-s001.pdf]

Supplementary Materials: Enhancement of Palmarumycin C12 and C13 Production by the Endophytic Fungus *Berkleasmium* sp. Dzf12 in an Aqueous-Organic Solvent System

Yan Mou, Dan Xu, Ziling Mao, Xuejiao Dong, Fengke Lin, Ali Wang, Daowan Lai, Ligang Zhou and Bingyan Xie

Table S1. Effects of seven organic solvents on mycelia growth in liquid culture of *Berkleasmium* sp. Dzf12.

Treatment	Mycelia biomass (g dw/L)						
	***n*-Dodecane**	***n*-Hexadecane**	**1-Hexadecene**	**Liquid Paraffin**	**Dibutyl Phthalate**	**Butyl Oleate**	**Oleic Acid**
CK	6.6 ± 0.6a	6.6 ± 0.4c	6.2 ± 0.4f	6.7 ± 0.6d	6.5 ± 0.6a	6.6 ± 0.5a	6.7 ± 0.3a
5% added on day 0	6.4 ± 0.4a	8.5 ± 0.6a	8.8 ± 0.3ab	8.8 ± 0.5ab	7.3 ± 0.4a	6.8 ± 0.3a	7.3 ± 0.4a
10% added on day 0	6.3 ± 0.3a	8.7 ± 0.4a	8.5 ± 0.6abcd	9.7 ± 0.5a	7.7 ± 0.2a	7.0 ± 0.3a	7.0 ± 0.7a
15% added on day 0	7.0 ± 0.2a	8.4 ± 0.2ab	7.5 ± 0.5cde	9.6 ± 0.4a	7.1 ± 0.2a	7.1 ± 0.4a	7.1 ± 0.4a
5% added on day 3	6.8 ± 0.8a	7.1 ± 0.4bc	9.2 ± 0.3ab	9.0 ± 0.2ab	7.7 ± 0.4a	6.7 ± 0.4a	7.0 ± 0.5a
10% added on day 3	6.3 ± 0.3a	7.6 ± 0.3abc	9.1 ± 0.3ab	9.2 ± 0.4ab	7.1 ± 0.7a	6.8 ± 0.7a	7.1 ± 0.5a
15% added on day 3	6.2 ± 0.5a	7.9 ± 0.4abc	8.1 ± 0.5bcde	8.3 ± 0.6abc	7.1 ± 0.5a	7.2 ± 0.4a	6.5 ± 0.5a
5% added on day 6	7.5 ± 0.4a	6.8 ± 0.4c	9.6 ± 0.2a	9.7 ± 0.5a	7.5 ± 0.1a	7.5 ± 0.3a	6.7 ± 0.5a
10% added on day 6	7.0 ± 0.6a	7.1 ± 0.2bc	9.0 ± 0.3ab	8.3 ± 0.2abc	7.1 ± 0.2a	7.0 ± 0.4a	6.4 ± 0.4a
15% added on day 6	6.8 ± 0.4a	6.6 ± 0.6c	9.0 ± 0.2ab	7.9 ± 0.3bcd	7.7 ± 0.3a	6.8 ± 0.2a	6.1 ± 0.3a
5% added on day 9	7.1 ± 0.4a	7.6 ± 0.1abc	8.9 ± 0.2ab	8.8 ± 0.4ab	7.0 ± 0.3a	6.9 ± 0.5a	6.4 ± 0.2a
10% added on day 9	6.7 ± 0.6a	7.3 ± 0.7abc	8.6 ± 0.6abc	8.8 ± 0.4ab	6.840.4a	6.8 ± 0.3a	6.2 ± 0.35a
15% added on day 9	7.0 ± 0.6a	7.1 ± 0.2bc	8.5 ± 0.4abcd	8.1 ± 0.5bcd	6.9 ± 0.3a	6.8 ± 0.2a	6.2 ± 0.2a
5% added on day 12	6.9 ± 0.9a	6.7 ± 0.2c	7.3 ± 0.4def	8.0 ± 0.3bcd	6.7 ± 0.5a	6.7 ± 0.3a	6.4 ± 0.6a
10% added on day 12	6.8 ± 0.3a	6.6 ± 0.3c	7.1 ± 0.6ef	6.8 ± 0.2d	6.6 ± 0.4a	6.7 ± 0.3a	6.8 ± 0.6a
15% added on day 12	6.0 ± 0.5a	6.6 ± 0.5c	7.0 ± 0.3ef	7.1 ± 0.5cd	6.6 ± 0.6a	6.8 ± 0.4a	6.6 ± 0.3a

Note: The organic solvents were applied at 5%, 10% and 15% on days 0, 3, 6, 9 and 12 of culture, respectively. The period of culture lasted for 15 days. "CK" means the control without any organic solvents. The values are expressed as means ± standard deviations (n = 3). Different letters indicate significant differences among the treatments in each column at $p \leq 0.05$.

Table S2. Effects of dibutyl phthalate on palmarumycin production in liquid culture of *Berkleasmium* sp. Dzf12.

Treatment	C12 Yield in Mycelia (mg/L)	C12 Yield in Aqueous Phase (mg/L)	C12 Yield in Organic Phase (mg/L)	C13 Yield in Mycelia (mg/L)	C13 Yield in Aqueous Phase (mg/L)	C13 Yield in Organic Phase (mg/L)	C12 Yield (mg/L)	C13 Yield (mg/L)	C12 Plus C13 Yield (mg/L)
CK	5.0 ± 0.6cd	nd	-	9.2 ± 1.0a	20.4 ± 1.9a	-	5.0	29.6	34.6
5% added on day 0	2.1 ± 0.5d	nd	148.5 ± 15.3a	0.0 ± 0.0e	nd	nd	150.6	0.0	150.6
10% added on day 0	5.6 ± 0.7cd	nd	100.8 ± 8.3bcde	0.0 ± 0.0e	nd	nd	106.4	0.0	106.4
15% added on day 0	2.5 ± 1.0d	nd	58.0 ± 16.2fg	0.0 ± 0.0e	nd	nd	60.5	0.0	60.5
5% added on day 3	5.2 ± 1.9cd	nd	129.7 ± 8.8ab	0.8 ± 0.1cde	nd	nd	134.9	0.8	135.6
10% added on day 3	6.1 ± 0.8cd	nd	107.2 ± 14.0bcd	0.7 ± 0.1cde	nd	nd	113.2	0.7	113.9
15% added on day 3	7.0 ± 1.3bcd	nd	87.1 ± 7.6cdef	0.7 ± 0.1cde	nd	nd	94.1	0.7	94.7
5% added on day 6	6.1 ± 0.9cd	nd	114.0 ± 6.5bc	0.5 ± 0.1de	nd	nd	120.1	0.5	120.5
10% added on day 6	11.3 ± 3.2ab	nd	77.2 ± 9.6def	0.8 ± 0.1cde	nd	nd	88.5	0.8	89.4
15% added on day 6	14.7 ± 2.0a	nd	58.6 ± 12.9fg	1.8 ± 0.2b	nd	nd	73.3	1.8	75.1
5% added on day 9	4.0 ± 0.7cd	nd	94.5 ± 10.8cde	0.0 ± 0.0e	nd	nd	98.4	0.0	98.5
10% added on day 9	8.7 ± 2.7bc	nd	68.8 ± 15.1efg	1.5 ± 0.1bc	nd	nd	77.5	1.5	79.0
15% added on day 9	4.5 ± 1.5cd	nd	40.3 ± 12.6g	0.2 ± 0.1e	nd	nd	44.7	0.2	44.9
5% added on day 12	3.6 ± 0.4cd	nd	52.7 ± 6.6fg	2.1 ± 0.3b	nd	nd	56.3	2.1	58.4
10% added on day 12	8.0 ± 1.4bc	nd	34.8 ± 8.3g	1.4 ± 0.2cde	nd	nd	42.8	1.4	44.2
15% added on day 12	3.8 ± 1.5cd	nd	34.1 ± 4.0g	0.2 ± 0.2e	nd	nd	37.9	0.2	38.0

Note: Dibutyl phthalate was applied at 5%, 10% and 15% on days 0, 3, 6, 9 and 12 of culture, respectively. The period of culture lasted for 15 days. "C12" means palmarumycin C_{12}, "C13" means palmarumycin C_{13}. "CK" means the control without any organic solvents. "-" means not applicable. "nd" means not detectable. The values are expressed as means ± standard deviations (n = 3). Different letters indicate significant differences among the treatments in each column at $p \leq 0.05$.

Table S3. Effects of butyl oleate on palmarumycin production in liquid culture of *Berkleasmium* sp. Dzf12.

Treatment	C12 Yield in Mycelia (mg/L)	C12 Yield in Aqueous Phase (mg/L)	C12 Yield in Organic Phase (mg/L)	C13 Yield in Mycelia (mg/L)	C13 Yield in Aqueous Phase (mg/L)	C13 Yield in Organic Phase (mg/L)	C12 Yield (mg/L)	C13 Yield (mg/L)	C12 Plus C13 Yield (mg/L)
CK	5.3 ± 2.0c	nd	-	7.6 ± 2.9a	24.6 ± 4.5a	-	5.3	32.1	37.5
5% added on day 0	3.5 ± 1.7c	3.2 ± 3.8bc	177.3 ± 9.5a	0.3 ± 0.0d	nd	nd	184.0	0.3	184.4
10% added on day 0	3.2 ± 0.9c	4.9 ± 1.0abc	132.3 ± 8.4bcd	0.2 ± 0.0d	nd	nd	140.4	0.2	140.6
15% added on day 0	4.1 ± 0.9c	3.6 ± 3.0bc	116.1 ± 12.5cde	0.1 ± 0.1d	nd	nd	123.8	0.1	123.9
5% added on day 3	23.2 ± 4.9ab	14.8 ± 3.3ab	153.5 ± 8.7ab	1.0 ± 0.2cd	nd	nd	191.6	1.0	192.5
10% added on day 3	34.2 ± 5.5a	16.9 ± 3.9a	139.4 ± 10.1bc	1.5 ± 0.4bcd	nd	nd	190.4	1.5	191.9
15% added on day 3	23.6 ± 7.0ab	16.7 ± 5.9a	127.6 ± 7.6bcd	0.8 ± 0.1d	nd	nd	167.8	0.8	168.6
5% added on day 6	17.1 ± 6.6bc	nd	136.3 ± 6.7bc	2.3 ± 0.4bcd	nd	nd	153.3	2.3	155.6
10% added on day 6	25.3 ± 4.8ab	nd	116.4 ± 7.1cde	2.1 ± 0.3bcd	nd	nd	141.8	2.1	143.9
15% added on day 6	21.4 ± 5.7ab	nd	82.5 ± 9.4f	0.9 ± 0.0cd	nd	nd	103.9	0.9	104.8
5% added on day 9	30.2 ± 5.4ab	nd	103.3 ± 5.9def	1.2 ± 0.1bcd	nd	nd	133.5	1.2	134.7
10% added on day 9	26.1 ± 6.0ab	nd	89.7 ± 17.2ef	1.6 ± 0.1bcd	nd	nd	115.8	1.6	117.3
15% added on day 9	24.8 ± 2.8ab	nd	73.0 ± 11.1f	1.9 ± 0.1bcd	nd	nd	97.7	1.9	99.7
5% added on day 12	19.2 ± 2.0abc	nd	34.9 ± 5.0g	3.6 ± 0.3b	nd	nd	54.1	3.6	57.7
10% added on day 12	21.9 ± 6.8ab	nd	31.0 ± 13.3g	3.4 ± 0.3bc	nd	nd	52.9	3.4	56.3
15% added on day 12	21.8 ± 5.9ab	nd	25.8 ± 8.6gh	3.6 ± 0.2b	nd	nd	47.6	3.6	51.2

Note: Butyl oleate was applied at 5%, 10% and 15% on days 0, 3, 6, 9 and 12 of culture, respectively. The period of culture lasted for 15 days. "C12" means palmarumycin C_{12}, "C13" means palmarumycin C_{13}. "CK" means the control without any organic solvents. "-" means not applicable. "nd" means not detectable. The values are expressed as means ± standard deviations (n = 3). Different letters indicate significant differences among the treatments in each column at $p \leq 0.05$.

Table S4. Effects of oleic acid on palmarumycin production in liquid culture of *Berkleasmium* sp. Dzf12.

Treatment	C12 Yield in Mycelia (mg/L)	C12 Yield in Aqueous Phase (mg/L)	C12 Yield in Organic Phase (mg/L)	C13 Yield in Mycelia (mg/L)	C13 Yield in Aqueous Phase (mg/L)	C13 Yield in Organic Phase (mg/L)	C12 Yield (mg/L)	C13 Yield (mg/L)	C12 Plus C13 Yield (mg/L)
CK	3.9 ± 1.2d	nd	-	8.5 ± 2.8a	25.3 ± 5.2a	-	3.9	33.8	37.8
5% added on day 0	22.4 ± 4.3bc	36.6 ± 4.6abc	125.6 ± 10.1a	0.1 ± 0.0b	nd	nd	184.6	0.1	184.7
10% added on day 0	32.8 ± 3.6ab	36.9 ± 6.8abc	93.6 ± 7.3bc	0.5 ± 0.1b	nd	nd	163.3	0.5	163.8
15% added on day 0	45.5 ± 8.1a	31.6 ± 3.4bcd	75.4 ± 5.5bcd	0.4 ± 0.1b	nd	nd	152.5	0.4	152.9
5% added on day 3	32.7 ± 5.2ab	26.2 ± 3.8cd	103.7 ± 11.2ab	0.3 ± 0.1b	nd	nd	162.6	0.3	162.9
10% added on day 3	24.4 ± 4.9bc	27.7 ± 4.9bcd	96.3 ± 13.2bc	0.6 ± 0.3b	nd	nd	148.4	0.6	148.9
15% added on day 3	25.3 ± 7.4bc	44.6 ± 7.8ab	85.6 ± 8.3bcd	0.1 ± 0.0b	nd	nd	155.4	0.1	155.5
5% added on day 6	14.9 ± 4.4bcd	39.5 ± 6.3abc	98.0 ± 10.2b	0.3 ± 0.1b	nd	nd	152.4	0.3	152.7
10% added on day 6	14.9 ± 3.7bcd	48.7 ± 4.1a	67.8 ± 6.8cd	0.1 ± 0.1b	nd	nd	131.3	0.1	131.5
15% added on day 6	17.2 ± 5.7bcd	37.2 ± 5.6abc	56.0 ± 10.2d	0.5 ± 0.2b	nd	nd	110.4	0.5	110.8
5% added on day 9	48.2 ± 7.6a	32.4 ± 4.0abcd	85.0 ± 7.5bcd	0.9 ± 0.3b	nd	nd	165.6	0.9	166.4
10% added on day 9	44.9 ± 5.1a	44.0 ± 6.2ab	83.1 ± 9.0bcd	1.5 ± 0.6b	nd	nd	172.0	1.5	173.5
15% added on day 9	44.7 ± 6.4a	34.7 ± 4.2abc	67.7 ± 11.0cd	1.0 ± 0.5b	nd	nd	147.0	1.0	148.0
5% added on day 12	12.5 ± 3.3cd	17.1 ± 2.9d	26.1 ± 6.1e	9.9 ± 2.3a	nd	nd	55.7	9.9	65.5
10% added on day 12	15.3 ± 4.2bcd	28.5 ± 4.7bcd	25.9 ± 7.3e	10.1 ± 3.0a	nd	nd	69.7	10.2	79.8
15% added on day 12	15.4 ± 5.2bcd	27.7 ± 4.4bcd	24.3 ± 8.5e	8.1 ± 2.5a	nd	nd	67.4	8.1	75.5

Note: Oleic acid was applied at 5%, 10% and 15% on days 0, 3, 6, 9 and 12 of culture, respectively. The period of culture lasted for 15 days. "C12" means palmarumycin C_{12}, "C13" means palmarumycin C_{13}. "CK" means the control without any organic solvents. "-" means not applicable. "nd" means not detectable. The values are expressed as means ± standard deviations (n = 3). Different letters indicate significant differences among the treatments in each column at $p \leq 0.05$.

Table S5. Effects of *n*-dodecane on palmarumycin production in liquid culture of *Berkleasmium* sp. Dzf12.

Treatment	C12 Yield in Mycelia (mg/L)	C12 Yield in Aqueous Phase (mg/L)	C12 Yield in Organic Phase (mg/L)	C13 Yield in Mycelia (mg/L)	C13 Yield in Aqueous Phase (mg/L)	C13 Yield in Organic Phase (mg/L)	C12 Yield (mg/L)	C13 Yield (mg/L)	C12 Plus C13 Yield (mg/L)
CK	5.8 ± 2.0bc	nd	-	6.5 ± 2.2d	23.7 ± 6.3abc	-	5.8	30.1	35.9
5% added on day 0	5.8 ± 2.3bc	nd	nd	23.6 ± 4.6bc	47.4 ± 10.2a	nd	5.8	71.0	76.7
10% added on day 0	5.9 ± 2.0bc	nd	nd	29.0 ± 5.2b	45.5 ± 8.2a	nd	5.9	74.4	80.3
15% added on day 0	7.2 ± 3.1bc	nd	nd	30.9 ± 3.2b	41.1 ± 6.2abc	nd	7.2	72.0	79.2
5% added on day 3	16.5 ± 4.9a	nd	nd	44.1 ± 9.4a	43.7 ± 5.4ab	nd	16.5	87.8	104.3
10% added on day 3	10.3 ± 4.0ab	nd	nd	10.1 ± 2.2cd	38.3 ± 12.1abc	nd	10.3	48.4	58.7
15% added on day 3	10.7 ± 2.7ab	nd	nd	11.4 ± 4.6cd	35.2 ± 7.9abc	nd	10.7	46.6	57.2
5% added on day 6	6.9 ± 2.1bc	nd	nd	17.7 ± 7.2bc	36.7 ± 6.6abc	nd	6.9	54.4	61.3
10% added on day 6	4.3 ± 2.0bc	nd	nd	7.0 ± 2.6d	31.7 ± 4.2abc	nd	4.3	38.7	43.0
15% added on day 6	3.9 ± 2.1bc	nd	nd	5.0 ± 2.0d	21.2 ± 8.3bc	nd	3.9	26.2	30.1
5% added on day 9	1.7 ± 0.9c	nd	nd	8.9 ± 2.9d	17.0 ± 6.2c	nd	1.7	25.9	27.6
10% added on day 9	1.3 ± 0.7c	nd	nd	8.5 ± 2.0d	19.3 ± 3.2c	nd	1.3	27.8	29.1
15% added on day 9	1.5 ± 1.0c	nd	nd	6.2 ± 3.0d	21.4 ± 4.0bc	nd	1.5	27.6	29.1
5% added on day 12	0.9 ± 0.1c	nd	nd	6.5 ± 2.3d	18.2 ± 8.8c	nd	0.9	24.7	25.5
10% added on day 12	1.0 ± 0.2c	nd	nd	9.1 ± 4.7d	17.9 ± 3.4c	nd	1.0	27.1	28.1
15% added on day 12	0.6 ± 0.1c	nd	nd	8.5 ± 4.1d	19.5 ± 4.2c	nd	0.6	28.0	28.6

Note: *n*-Dodecane was applied at 5%, 10% and 15% on days 0, 3, 6, 9 and 12 of culture, respectively. The period of culture lasted for 15 days. "C12" means palmarumycin C_{12}, "C13" means palmarumycin C_{13}. "CK" means the control without any organic solvents. "-" means not applicable. "nd" means not detectable. "0-1" means that the solvent was applied at 5% on day 0. The values are expressed as means ± standard deviations (n = 3). Different letters indicate significant differences among the treatments in each column at $p \leq 0.05$.

Table S6. Effects of *n*-hexadecane on palmarumycin production in liquid culture of *Berkleasmium* sp. Dzf12.

Treatment	C12 Yield in Mycelia (mg/L)	C12 Yield in Aqueous Phase (mg/L)	C12 Yield in Organic Phase (mg/L)	C13 Yield in Mycelia (mg/L)	C13 Yield in Aqueous Phase (mg/L)	C13 Yield in Organic Phase (mg/L)	C12 Yield (mg/L)	C13 Yield (mg/L)	C12 Plus C13 Yield (mg/L)
CK	4.6 ± 1.6c	0.0 ± 0.0c	-	10.6 ± 3.2a	21.2 ± 6.2cde	-	4.6	31.8	36.4
5% added on day 0	19.4 ± 5.2abc	8.1 ± 2.2bc	nd	2.3 ± 0.9b	42.9 ± 6.1ab	nd	27.5	45.2	72.7
10% added on day 0	25.9 ± 7.5ab	15.8 ± 5.2ab	nd	4.0 ± 1.3b	37.2 ± 9.7abcd	nd	41.7	41.2	82.9
15% added on day 0	16.9 ± 4.2bc	15.9 ± 4.7ab	nd	3.8 ± 1.6b	34.2 ± 4.8abcd	nd	32.8	38.0	70.7
5% added on day 3	17.6 ± 4.3bc	11.6 ± 3.1abc	nd	4.8 ± 1.0ab	46.8 ± 7.2a	nd	29.2	51.6	80.8
10% added on day 3	24.1 ± 6.8abc	12.2 ± 4.2abc	nd	7.2 ± 2.9ab	39.3 ± 5.5abc	nd	36.3	46.4	82.7
15% added on day 3	17.6 ± 3.9bc	9.4 ± 2.6abc	nd	6.5 ± 2.1ab	35.2 ± 6.2abcd	nd	27.0	41.7	68.6
5% added on day 6	23.8 ± 5.7abc	16.9 ± 5.4ab	nd	5.0 ± 2.1ab	36.7 ± 5.3abcd	nd	40.7	41.7	82.4
10% added on day 6	18.9 ± 4.9bc	6.1 ± 2.2bc	nd	4.9 ± 1.8ab	23.3 ± 8.8bcde	nd	25.0	28.2	53.1
15% added on day 6	18.2 ± 4.7bc	4.9 ± 1.7bc	nd	4.5 ± 2.0ab	22.6 ± 5.4bcde	nd	23.1	27.1	50.1
5% added on day 9	37.7 ± 8.7ab	9.5 ± 2.7abc	nd	2.3 ± 0.9b	18.2 ± 4.0de	nd	47.2	20.5	67.7
10% added on day 9	39.8 ± 7.6a	10.7 ± 3.1abc	nd	7.8 ± 2.0ab	17.5 ± 8.1de	nd	50.5	25.2	75.7
15% added on day 9	31.0 ± 9.0ab	22.3 ± 6.2a	nd	4.0 ± 1.3b	11.4 ± 6.2e	nd	53.3	15.3	68.6
5% added on day 12	35.3 ± 7.1ab	11.2 ± 2.3abc	nd	1.8 ± 1.0b	10.1 ± 4.6e	nd	46.5	11.9	58.5
10% added on day 12	37.2 ± 4.2ab	13.1 ± 3.8ab	nd	7.1 ± 2.0ab	8.4 ± 3.0e	nd	50.3	15.5	65.7
15% added on day 12	27.1 ± 6.1ab	16.0 ± 5.4ab	nd	3.8 ± 1.0b	7.4 ± 2.7e	nd	43.1	11.2	54.3

Note: *n*-Hexadecane was applied at 5%, 10% and 15% on days 0, 3, 6, 9 and 12 of culture, respectively. The period of culture lasted for 15 days. "C12" means palmarumycin C_{12}, "C13" means palmarumycin C_{13}. "CK" means the control without any organic solvents. "-" means not applicable. "nd" means not detectable. The values are expressed as means ± standard deviations (n = 3). Different letters indicate significant differences among the treatments in each column at $p \leq 0.05$.

Table S7. Effects of 1-hexadecene on palmarumycin production in liquid culture of *Berkleasmium* sp. Dzf12.

Treatment	C12 Yield in Mycelia (mg/L)	C12 Yield in Aqueous Phase (mg/L)	C12 Yield in Organic Phase (mg/L)	C13 Yield in Mycelia (mg/L)	C13 Yield in Aqueous Phase (mg/L)	C13 Yield in Organic Phase (mg/L)	C12 Yield (mg/L)	C13 Yield (mg/L)	C12 Plus C13 Yield (mg/L)
CK	6.5 ± 2.9ab	nd	-	7.8 ± 3.0fg	19.8 ± 6.2de	-	6.5	27.6	34.1
5% added on day 0	3.6 ± 1.7ab	nd	nd	13.3 ± 3.2efd	21.1 ± 5.8de	nd	3.6	34.4	38.0
10% added on day 0	2.7 ± 1.0ab	nd	nd	14.5 ± 3.1defg	25.5 ± 6.1cde	nd	2.7	39.9	42.5
15% added on day 0	1.9 ± 1.0b	nd	nd	7.1 ± 2.1g	19.5 ± 3.3de	nd	1.9	26.7	28.5
5% added on day 3	3.3 ± 1.9ab	nd	nd	24.3 ± 5.6bcdef	49.4 ± 9.1bc	nd	3.3	73.7	77.1
10% added on day 3	3.2 ± 1.6ab	nd	nd	25.2 ± 6.0bcde	53.8 ± 11.0b	nd	3.2	79.0	82.2
15% added on day 3	2.4 ± 1.4ab	nd	nd	11.9 ± 3.2efg	14.0 ± 3.8de	nd	2.4	25.9	28.4
5% added on day 6	3.3 ± 2.3ab	nd	nd	33.7 ± 6.2abc	93.4 ± 12.6a	nd	3.3	127.1	130.4
10% added on day 6	2.5 ± 1.2ab	nd	nd	37.2 ± 7.4ab	92.9 ± 14.0a	nd	2.5	130.1	132.6
15% added on day 6	6.0 ± 2.6ab	nd	nd	11.8 ± 3.8efg	27.4 ± 4.6cde	nd	6.0	39.3	45.3
5% added on day 9	4.7 ± 1.9ab	nd	nd	42.7 ± 9.5a	89.6 ± 10.8a	nd	4.7	132.3	137.0
10% added on day 9	6.9 ± 2.7ab	nd	nd	30.9 ± 5.5abcd	38.1 ± 6.6bcd	nd	6.9	69.0	75.8
15% added on day 9	4.8 ± 1.5ab	nd	nd	20.7 ± 4.1bcdefg	13.9 ± 3.3de	nd	4.8	34.5	39.3
5% added on day 12	8.9 ± 2.9ab	nd	nd	20.2 ± 4.6cdefg	24.0 ± 4.2de	nd	8.9	44.2	53.1
10% added on day 12	9.7 ± 3.1a	nd	nd	19.5 ± 3.4cdefg	21.3 ± 4.2de	nd	9.7	40.8	50.4
15% added on day 12	7.1 ± 2.6ab	nd	nd	14.6 ± 2.6defg	9.9 ± 3.3e	nd	7.1	24.5	31.6

Note: 1-Hexadecene was applied at 5%, 10% and 15% on days 0, 3, 6, 9 and 12 of culture, respectively. The period of culture lasted for 15 days. "C12" means palmarumycin C_{12}, "C13" means palmarumycin C_{13}. "CK" means the control without any organic solvents. "-" means not applicable. "nd" means not detectable. The values are expressed as means ± standard deviations (n = 3). Different letters indicate significant differences among the treatments in each column at $p \leq 0.05$.

Table S8. Effects of liquid paraffin on palmarumycin production in liquid culture of *Berkleasmium* sp. Dzf12.

Treatment	C12 Yield in Mycelia (mg/L)	C12 Yield in Aqueous Phase (mg/L)	C12 Yield in Organic Phase (mg/L)	C13 Yield in Mycelia (mg/L)	C13 Yield in Aqueous Phase (mg/L)	C13 Yield in Organic Phase (mg/L)	C12 Yield (mg/L)	C13 Yield (mg/L)	C12 Plus C13 Yield (mg/L)
CK	6.0 ± 3.0a	nd	-	8.8 ± 3.5d	22.0 ± 8.2c	-	6.0	30.8	36.9
5% added on day 0	10.5 ± 3.6a	nd	nd	43.9 ± 9.8abc	77.9 ± 7.1a	nd	10.5	121.8	132.2
10% added on day 0	10.0 ± 3.9a	nd	nd	52.5 ± 6.1a	80.3 ± 12.3a	nd	10.0	132.7	142.8
15% added on day 0	9.2 ± 3.0a	nd	nd	47.8 ± 10.2ab	84.7 ± 9.3a	nd	9.2	132.4	141.6
5% added on day 3	12.3 ± 4.1a	nd	nd	40.6 ± 6.6abc	78.4 ± 7.9a	nd	12.3	119.0	131.3
10% added on day 3	11.2 ± 3.8a	nd	nd	51.1 ± 8.3a	83.0 ± 5.2a	nd	11.2	134.1	145.3
15% added on day 3	10.7 ± 3.2a	nd	nd	36.6 ± 4.8abc	67.1 ± 9.7ab	nd	10.7	103.7	114.4
5% added on day 6	7.5 ± 2.7a	nd	nd	47.3 ± 9.5abc	83.4 ± 9.2a	nd	7.5	130.6	138.2
10% added on day 6	6.3 ± 2.9a	nd	nd	32.8 ± 6.1abc	87.8 ± 8.3a	nd	6.3	120.6	126.9
15% added on day 6	3.7 ± 1.9a	nd	nd	30.2 ± 5.6abcd	73.6 ± 7.0a	nd	3.7	103.9	107.6
5% added on day 9	4.4 ± 1.8a	nd	nd	26.0 ± 5.3bcd	60.3 ± 7.4ab	nd	4.4	86.2	90.7
10% added on day 9	5.3 ± 2.1a	nd	nd	29.8 ± 7.1abcd	67.7 ± 11.4ab	nd	5.3	97.6	102.9
15% added on day 9	6.1 ± 2.9a	nd	nd	32.0 ± 2.1abcd	62.5 ± 5.2ab	nd	6.1	94.4	100.5
5% added on day 12	7.7 ± 2.8a	nd	nd	23.4 ± 6.4cd	41.9 ± 8.9bc	nd	7.7	65.3	73.0
10% added on day 12	6.9 ± 2.8a	nd	nd	32.5 ± 8.5abc	32.3 ± 6.3c	nd	6.9	64.8	71.7
15% added on day 12	3.8 ± 1.9a	nd	nd	27.1 ± 5.7bcd	33.3 ± 5.5c	nd	3.8	60.4	64.2

Note: Liquid paraffin was applied at 5%, 10% and 15% on days 0, 3, 6, 9 and 12 of culture, respectively. The period of culture lasted for 15 days. "C12" means palmarumycin C_{12}, "C13" means palmarumycin C_{13}. "CK" means the control without any organic solvents. "-" means not applicable. "nd" means not detectable. The values are expressed as means ± standard deviations (n = 3). Different letters indicate significant differences among the treatments in each column at $p \leq 0.05$.

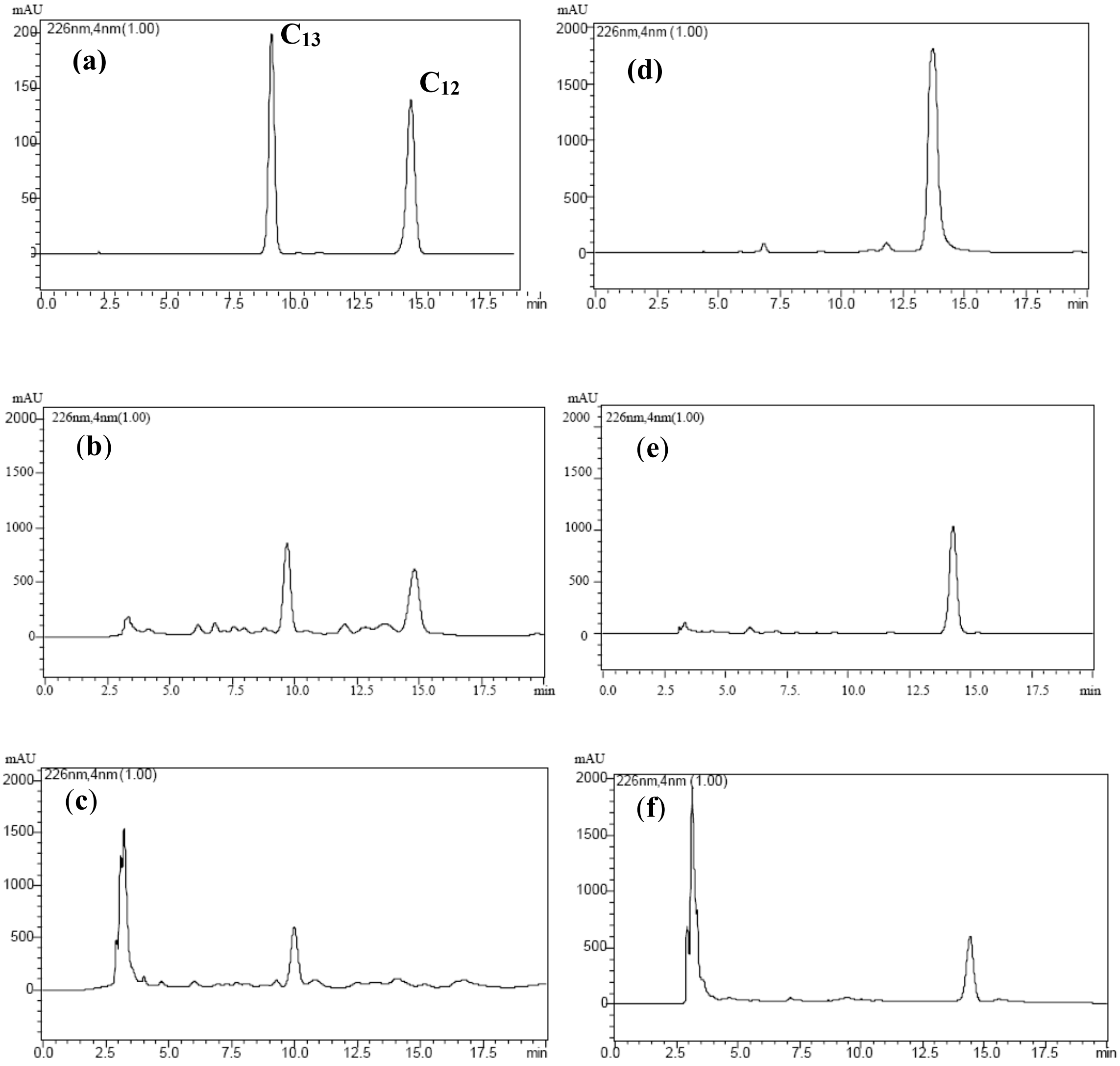

Figure S1. HPLC analysis of palmarumycin production in liquid culture of *Berkleasmium* sp. Dzf12 with oleic acid as the water-immiscible organic solvent. (**a**): Palmarumycins C_{12} and C_{13}; (**b**): Mycelia extract without addition of oleic acid; (**c**): Broth extract without addition of oleic acid; (**d**): Extract of oleic acid phase; (**e**): Mycelia extract with addition of oleic acid; (**f**): Broth extract with addition of oleic acid.
